# Experimental Myocardial Infarction Induces Altered Regulatory T Cell Hemostasis, and Adoptive Transfer Attenuates Subsequent Remodeling

**DOI:** 10.1371/journal.pone.0113653

**Published:** 2014-12-01

**Authors:** Rinat Sharir, Jonathan Semo, Sara Shimoni, Tamar Ben-Mordechai, Natalie Landa-Rouben, Sofia Maysel-Auslender, Aviv Shaish, Michal Entin–Meer, Gad Keren, Jacob George

**Affiliations:** 1 Heart Center, Kaplan Medical Center, Rehovot, Israel, Affiliated to the Hebrew University, Jerusalem, Israel; 2 Laboratory of Cardiovascular Research, Department of Cardiology, Tel-Aviv Sourasky Medical Center, Tel-Aviv, Israel; 3 Sackler Faculty of Medicine, Tel Aviv University, Tel Aviv, Israel; 4 Neufeld Cardiac Research Institute, Tel Aviv University, Sheba Center for Regenerative Medicine, Stem Cells, and Tissue Engineering and Tamman Cardiovascular Research institute, Ramat-Gan, Israel; 5 The Bert W. Strassburger Lipid Center, Sheba Medical Center, Tel-Hashomer, Ramat-Gan, Israel; Uniform Services University of the Health Sciences, United States of America

## Abstract

**Background:**

Ischemic cardiac damage is associated with upregulation of cardiac pro-inflammatory cytokines, as well as invasion of lymphocytes into the heart. Regulatory T cells (Tregs) are known to exert a suppressive effect on several immune cell types. We sought to determine whether the Treg pool is influenced by myocardial damage and whether Tregs transfer and deletion affect cardiac remodeling.

**Methods and Results:**

The number and functional suppressive activity of Tregs were assayed in mice subjected to experimental myocardial infarction. The numbers of splenocyte-derived Tregs in the ischemic mice were significantly higher after the injury than in the controls, and their suppressive properties were significantly compromised. Compared with PBS, adoptive Treg transfer to mice with experimental infarction reduced infarct size and improved LV remodeling and functional performance by echocardiography. Treg deletion with blocking anti-CD25 antibodies did not influence infarct size or echocardiographic features of cardiac remodeling.

**Conclusion:**

Treg numbers are increased whereas their function is compromised in mice with that underwent experimental infarction. Transfer of exogeneous Tregs results in attenuation of myocardial remodeling whereas their ablation has no effect. Thus, Tregs may serve as interesting potential interventional targets for attenuating left ventricular remodeling.

## Introduction

Heart failure is a frequent cause of death in the industrialized world [1]–[2]. Approximately 6 million people suffer from heart failure in the United States alone, resulting in about 300,000 deaths per year [3]. The major cause of heart failure is myocardial infarction caused by atherothrombotic epicardial coronary arterial obstruction [4]–[6].

Heart failure following myocardial infarction can result from a substantial loss of cardiomyocytes in the infarcted zone, but more often is precipitated by delayed and progressive pathological remodeling of the left ventricle (LV). When myocardial tissue is injured, a normal healing response is initiated through a series of complex events that include acute inflammation, formation of granulation tissue, and eventual scar formation [7]–[8]. Cytokines and growth factors are released to recruit white blood cells, mainly neutrophils. Monocytes are then recruited to the wound site, where they differentiate into macrophages. The macrophages are responsible for clearing the infarcted zone and also for recruiting cells such as fibroblasts, endothelial cells and stem/progenitor cells, with consequent formation of granulation tissue. Blood vessel formation is essential for healing of the infarcted myocardium. Granulation tissue is subsequently replaced by extracellular matrix (ECM), which is deposited primarily by fibroblasts and remodeled into scar tissue [9].

The concept of ‘suppressor’ T cells acting to down regulate the host's immune system arose as long ago as the early 1970s [10]–[11]. The naturally occurring population of CD4^+^CD25^+^ T cells (regulatory T cells; Tregs), both in naïve mice and in humans, constitutes 5–10% of the peripheral CD4^+^ T cells and less than 1% of the peripheral CD8^+^ T cells [12]. A previous study by our group showed that CD4^+^CD25^+^ Tregs may play a protective role in the progression of atherosclerosis and in patients with acute coronary syndromes [13]. We also demonstrated that in these conditions the numbers of naturally occurring CD4^+^CD25^+^ Tregs are reduced and their functional properties are compromised [14].

It has been reported that insufficient recruitment of Tregs via the CCR5 receptor results in worsening of ventricular remodeling [15]. A recent study described a role for Tregs in a rat model of myocardial infarction [16], and in a study in mice it was shown that CD4^+^ T-cells become activated after myocardial infarction and facilitate wound healing of the myocardium [17].

In this study we show for the first time that Tregs become dysfunctional after experimental myocardial infarction, whereas their numbers increase. Moreover, whereas adoptive transfer of Tregs attenuates remodeling, their ablation with blocking antibodies does not influence this process.

## Methods

### Ethical Statement

The study was performed in accordance with the guidelines of The Animal Care and Use Committee of Sheba Medical Center, Tel-Aviv University, which conforms to the policies of the American Heart Association and the Guide for the Care and Use of Laboratory Animals. The experiment was conducted with the approval of the ethics committee of the University of Tel-Aviv (IACUC under protocol number M-09-076).

### Animals

Mature male C57BL/6 mice, 10–12 weeks old, weighing 20–25 g were purchased from Harlan Laboratories, Jerusalem.

### Surgical procedure

Myocardial infarction was induced in the mice by permanent ligation of the left anterior descending coronary artery (LAD) (*n*  =  10–20 per group). Mice were anesthetized with 2% isoflurane, intubated orally, and artificially ventilated with a respirator. A small oblique thoracotomy was performed lateral to the midsternal line in the third intercostal space to expose the heart. The pericardium was opened and the proximal left anterior descending artery branch of the left coronary artery was ligated. Sham-operated mice underwent the same surgical procedure without ligation of the artery and served as controls. Dypirone 60 mg/kg subcutaneous (s.c) injection was given after induction of the myocardial infarction.

For measurements of kinetics, mice from each group (ischemic mice (n = 18) and sham- operated mice (n = 20)) were sacrificed by carbon dioxide (CO_2_) inhalation, using gradual filling of the chamber, 1 day (n = 8), 5 days (n = 7), 14 days (n = 13), or 30 days (n = 13) after LAD ligation. For the adoptive transfer experiment and for the depletion experiment, mice were anaesthetized by intraperitoneal (i.p) injection of an overdose of Phenobarbital sacrificed 28 days following the procedure.

### Cell separation and flow cytometry

At the above mentioned time points after LAD ligation, organs (spleens and hearts) were collected for flow cytometric (FACS) analysis and functional suppression assay. Splenocytes were co-stained with the following monoclonal antibodies: fluorescein isothiocyanate-labeled anti-CD4 (GK1.5; Miltenyi Biotec, Bergisch Gladbach, Germany), phycoerythrin-labeled anti-CD25 (7D4; Miltenyi Biotec), and allophycocyanin anti-mouse/rat forkhead box P3 (FOXP3) (clone FJK-16s; eBioscience, San Diego, CA). Flow cytometry was performed with a BD FACSCanto II flow cytometer, for the adoptive transfer assay, cells were sorted with a FACSAria II device, and the results were analyzed by FACSDiva software (all from Becton Dickinson, Franklin Lakes, NJ). For Tregs depletion, splenocytes were isolated 7 days post ischemia and were co-stained with the Tregs markers.

### Co-culture of effector and Tregs

For co-culturing of CD4^+^CD25^-^ T cells (effector T cells; Teffs) and Tregs (n = 8–10), 96-well plates were coated with 1 µg/ml of anti-CD3 monoclonal antibody (clone 145-2C11, eBioscience) overnight at 4°C. Teff as responders and CD4^+^CD25^hi^ Tregs as suppressors (2×10^4^ cells/well) were co-cultured in RPMI medium supplemented with 10% fetal calf serum at different ratios of suppressors to responders (1∶1, 1∶2, 1∶4). All cells were seeded in a final volume of 200 µl in the presence of soluble anti-mouse CD28 (clone 37.51, eBioscience). After 72 hours, [^3^H] thymidine (1 µCi/well) was added for 18 hours, and cell proliferation was then assayed by scintillation counting in a beta counter. Percent inhibition of proliferation was determined with the following formula: 1-(median [^3^H] thymidine uptake of 1∶1 responder/suppressor co-culture/median [^3^H]thymidine uptake of Treg only).

### Study design for in-vivo adoptive transfer

One day after induction of myocardial infarction, mice received 2×10^5^ purified CD4^+^CD25^hi^ Tregs (n  =  15) or equal volumes of phosphate-buffered saline (PBS) as control (n  =  15), injected once into the tail vein. After exclusion of mice that died during induction of anesthesia or myocardial infarction, the final analysis was done on 14 mice, which were then sacrificed on day 28.

### Treg ablation with Anti-CD25 Monoclonal Antibody

One day prior induction of LAD ligation and 14 days after the procedure, 200 µg of anti-CD25 (clone PC61) was administered intraperitoneally in a volume of 200 µl sterile saline (n = 15). Rat IgG1 K isotype control functional grade purified (clone 16-4301, eBioscience) was used as control at the same volume (n = 12). The extent of Treg depletion was evaluated by a prior study using different concentration of anti-CD25 given via the tail vein [18]. The depleting antibody had a different epitope than used in the flow cytometric assay [19].

### Detection of injected Tregs labeled with DiI on frozen heart sections

To determine whether the injected cells home to the injured myocardium, one day after induction of myocardial infarction, we injected mice intravenously with Tregs labeled with *1,1' - Dioctadecyl - 3,3,3',3' - tetramethylindocarbocyanine iodide* (DiI). For control sham operated mice were injected with labeled Tregs (n = 8) in addition, mice that underwent myocardial infarction were injected with labeled non-Tregs (n = 8). Hearts were then harvested on days 1(n = 8), 4 (n = 10), and 7(n = 8) after the injection, control mice were sacrificed at day 4 post injection, the hearts were sectioned into two transverse slices parallel to the atrioventricular ring, embedded in OCT and frozen immediately. The OCT blocks were cryosectioned into 5-µm slices, fixed with 70% ethanol, stained for double-stranded DNS with 4',6-diamidino-2-phenylindole (DAPI), covered, and then examined under a fluorescence microscope (Olympus BX 51).

### Echocardiography

Mice were anesthetized as described above. Transthoracic echocardiography was performed with a mouse echocardiography system (Vevo 770, VisualSonics, Toronto, Canada) equipped with a 35 MHz phased array. Recordings were obtained from all mice (Tregs or PBS injection) 1 day after the myocardial infarction (baseline echocardiogram) and 4 weeks after myocardial infarction. Hearts were imaged two-dimensionally in the parasternal long- and short-axis views, through which the M-mode cursor was positioned perpendicular to the left ventricle (LV) septum and posterior wall [20]–[21]. All measurements were performed by an experienced technician blinded to the treatment group, and were averaged for three consecutive cardiac cycles.

### Histological and heart morphometric analysis

Hearts were arrested with 15% KCl, perfused with 4% formaldehyde (15 mm Hg) for 20 minutes and cut into two transverse slices parallel to the atrioventricular ring. Each slice was fixed with 4% buffered formalin, embedded in paraffin, and sectioned into 5-µm slices. Serial sections were stained with either hematoxylin and eosin to detect cellular infiltration or with Masson's trichrome stain (Sigma, St Louis, MO) to detect fibrosis and assess its area. To assess vessel density (mean number of capillaries and arterioles/mm2), five adjacent fields (at x 400 magnification) of each section were examined at the border between the viable myocardium and the scar and stained against CD31 (Santa Cruz Biotechnology, CA, USA).

All slides were digitally photographed, analyzed with manual planimetry software (Sigma Scan Pro version 5; SPSS; Chicago, IL), and then used to evaluate LV remodeling as previously described [20]–[21].

### Statistical analysis

GraphPad Prism, version 5.00 for Windows (GraphPad Software, San Diego, CA), was used for statistical analysis. All variables are expressed as means ± SEM. The Mann-Whitney test (if data were not normally distributed) or unpaired t-test (if data were normally distributed) were used to compare between two groups. Changes in FACS-derived measurements in mice that underwent myocardial infarction and in sham-operated mice were compared by repeated measures two-way ANOVA followed by the Mann-Whitney test. Differences between baseline and 30 days were assessed using 2-tail paired t tests. To test the hypothesis that changes in measures of LV function between 1 and 30 days varied among the experimental groups, a general linear model 2-way repeated-measures ANOVA was used. The model included the effects of treatment, time, and treatment-by-time interaction. The Bonferroni correction was used to assess the significance of predefined comparisons at specific time points.In addition, relative change (%) in baseline parameters were calculated as [(follow-up parameter minus baseline parameter)/baseline parameter] × 100, and were assessed by means of two-tailed unpaired t tests. To assess the number of DiI-labeled Tregs, data were compared by Kruskal-Wallis with Dunn's multiple comparisons.

## Results

### Percentages of Tregs in lymphoid tissue is increased after myocardial infarction

For the quantitative assay, 40 mice were included in the experiment; two mice died during induction of anesthesia or myocardial infarction. The final analysis was performed with 18 mice for the MI group and 20 mice for control.

**Figure 1:**
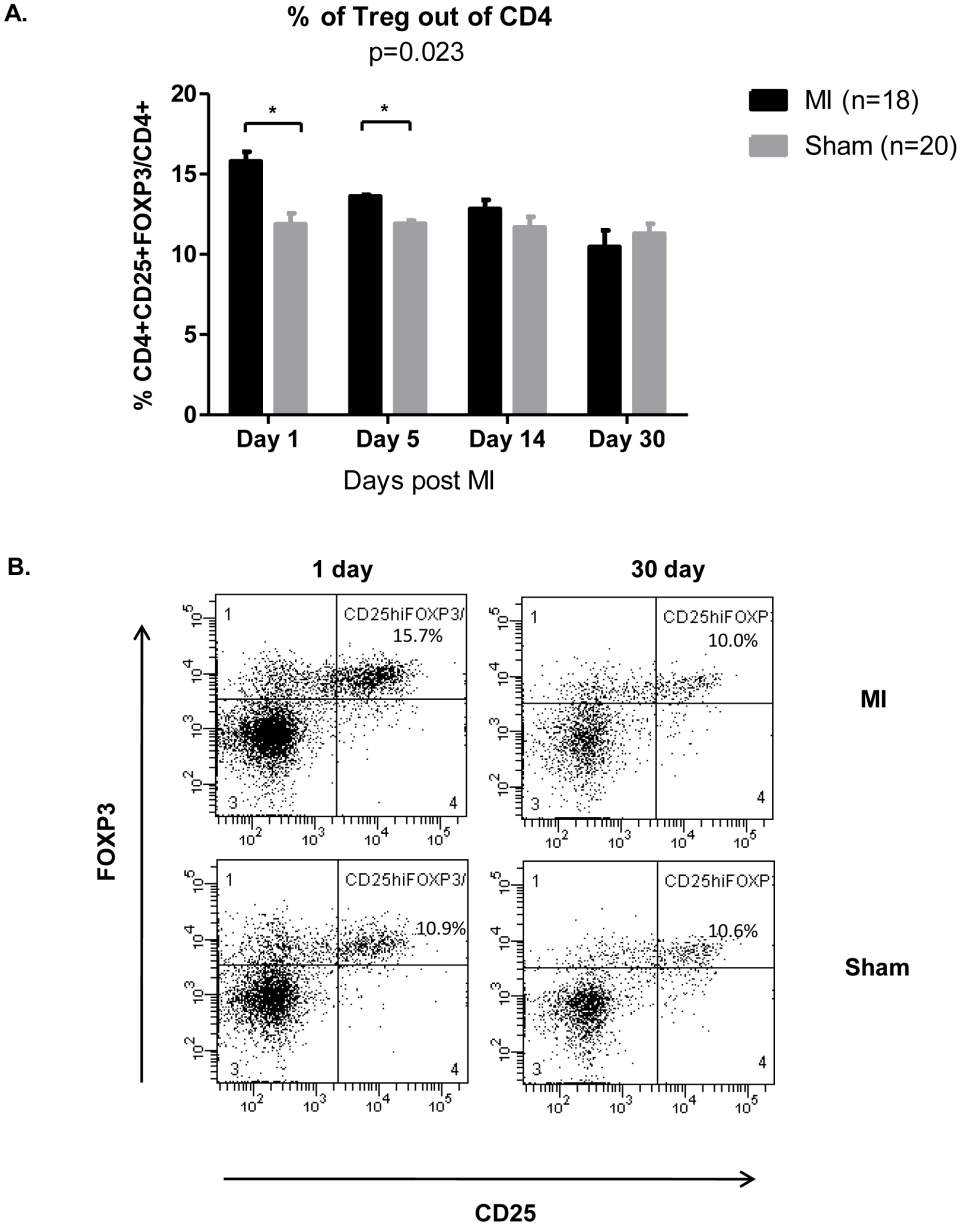
Kinetics of spleen-derived Tregs. The Treg population is increased after experimental myocardial infarction. (A) FACS assay of Tregs after myocardial infarction shows the numbers of Tregs, expressed as a percentage of the total number of CD4 cells, 1 day, 5 days, 14 days and 30 days after myocardial infarction. Treg cell measured by CD4^+^CD25^+^FOXP3 (B) Representative FACS results showing kinetics of Tregs 1 day and 30 days after experimental myocardial infarction. p based on Two-way ANOVA; * p<0.05 based on Mann-Whitney test. n (MI) = 18; n(sham) = 20

To determine whether Treg pool is altered after experimental infarction we first evaluated the kinetics of Tregs in the spleen. We chose four different time points after myocardial infarction and measured the levels of CD4^+^CD25^+^FOXP3 out of the total splenic CD4 population. During the first 24 hours after myocardial infarction, Treg numbers in the spleens of ischemic mice were significantly increased (15.8 ± 0.58%) compared with sham-operated mice (11.88 ± 0.68%) (*p* <0.03; Fig. 1A). Treg numbers then decreased, and by day 30 after myocardial infarction, their levels in the ischemic mice were similar to those of the sham-operated control mice (day 5, 13.6 ± 0.1% in ischemic mice vs. 11.8 ± 0.3% in controls, p<0.05; day 14, 12.82 ± 0.6% in ischemic mice vs. 11.68 ± 0.6% in controls; day 30, 10.46 ± 1.0% in ischemic mice vs. 11.3 ± 0.6% in controls). At all experimental time points the levels of CD4^+^CD25^+^FOXP3 in the sham-operated mice group were similar (approximately 10.5%; Fig. 1A). A representative FACS recording shows the Treg percentages of ischemic vs. sham-operated mice on day 1 post myocardial infarction compared to their percentages on day 30 post myocardial infarction (Fig. 1B).

### Tregs are present in the injured heart tissue after myocardial infarction

**Figure 2:**
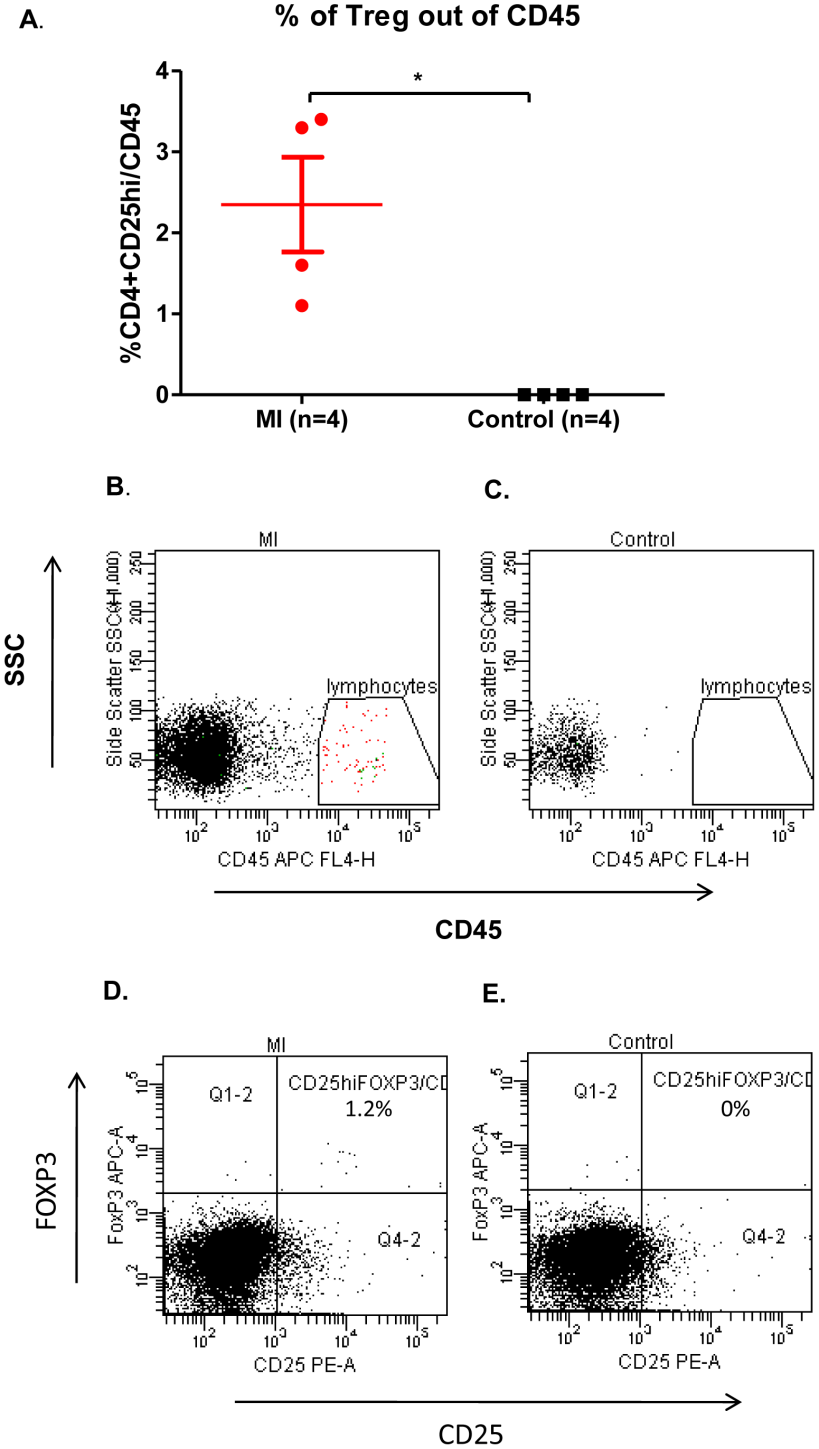
Tregs migrate to the injured heart. (A) FACS analysis records the numbers of Tregs (CD4^+^CD25^hi^ cells) expressed as a percentage of the total number of CD45 cells after myocardial infarction. (B–C) Representative FACS recordings of the kinetics of CD45 presence in mice with experimentally induced myocardial infarction compared with control mice. (D–E) Representative FACS pictures of CD25^+^FOXP3 out of CD4 in infarct heart or control heart. *p<0.03 based on Mann-Whitney test, n =  4;

It is well established that there are no lymphocytes present in the healthy heart [22]. After induction of myocardial infarction, hearts were enzymatically dissociated into single cell suspensions and analyzed by FACS. Seven days after myocardial infarction the mouse hearts clearly demonstrated the presence of CD4^+^CD25^hi^ lymphocytes (Fig. 2B). We found a significant increase in the percentage of Tregs out of CD45 positive cells (Fig. 2A, *p* <0.03) following myocardial infarction induction. Figure 2B and 2C shows representative FACS recordings of side scatter stained by CD45 at myocardial infarction group vs. control group, while figure 2D and 2E shows CD25^+^ FOXP3 cells expressed as percentages of CD4 in ischemic vs. control mice.

### Suppressive properties of Tregs are compromised after experimental myocardial infarction

**Figure 3:**
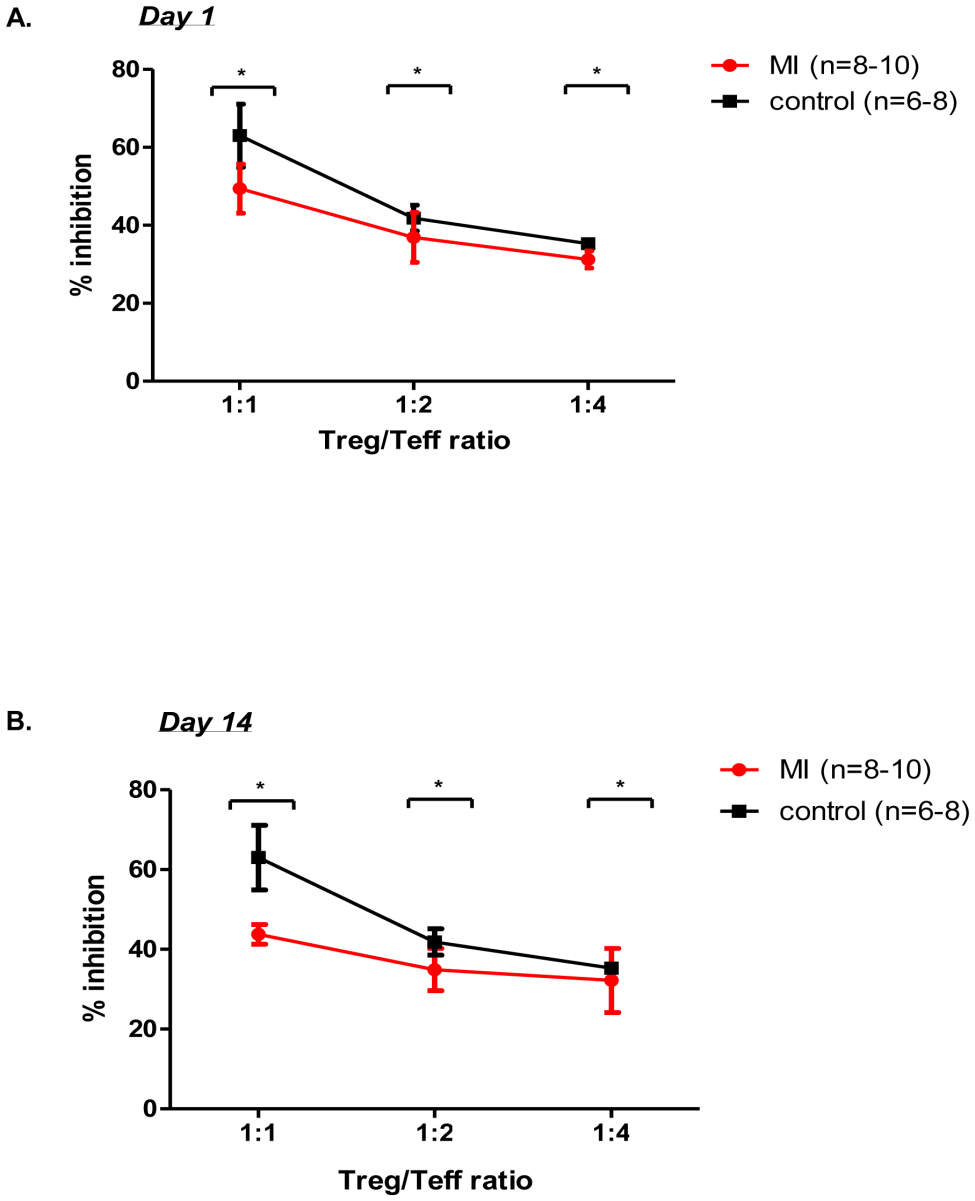
Suppressive properties of Tregs. Assay of functional suppression assay (A–B) shows a trend towards decline in the suppressive properties of Tregs, with significant effects at two time points after experimental induction of myocardial infarction at all ratios. p based on Two-way ANOVA; * p<0.05 based on Mann-Whitney test, n =  6–10;

To assess the suppressive properties of Tregs from mice with induced myocardial infarction, we used an *in-vitro* functional suppression assay, which we performed at two selected time points (Fig. 3), equivalent to those chosen for the kinetic Treg assay: day 1 (Fig. 3A) and day 14 (Fig. 3B). We have found that the functional suppressive properties of mice after experimental myocardial infarction are reduced as compared with those from control animals We used T-test for each of the individual ratios and the results were presented that were found significant.

### Fluorescently labeled regulatory T cells migrate to the injured heart

**Figure 4:**
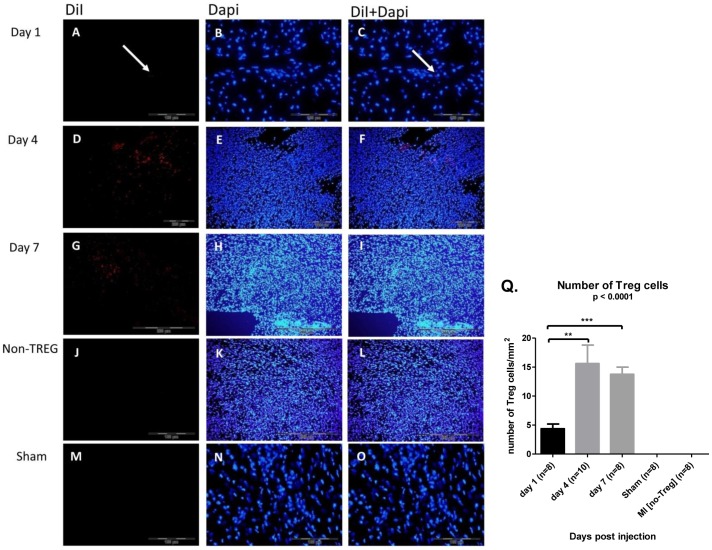
Homing of Tregs to ischemic hearts after their intravenous injection. (A–C) Frozen heart sections 1 day after intravenous (i.v.) injection of DiI-labeled Tregs. (D–F) Frozen heart sections 4 days after i.v. injection of DiI-labeled Tregs. (G–I) Frozen heart sections 7 days after i.v. injection of DiI-labeled Tregs. (J–L) Frozen heart sections 4 days after i.v. injection of DiI-labeled no-Tregs. (M–O) Frozen heart sections 4 days after i.v. injection of DiI-labeled Tregs sham mice. (Q) Treg numbers are significantly increased by day 4 post i.v. injection. p based on Kruskal-Wallis; **p<0.001, ***p<0.0001 based on Dunn's multiple comparison test. n (day 1) = 8; n (day 4) = 10; n (day 7) = 8, MI no-Treg cells (n = 8), Sham (n = 8).

To study whether Tregs migrate to the injured heart, DiI-labeled Tregs were systemically injected into mice 1 day after myocardial infarction. In mice that were sacrificed 1 day, 4 days and 7 days after injection, DiI-stained Tregs were observed in the infarcted hearts. Tregs with the DiI label are shown in Fig. 4A, 4D, 4G, 4J and 4M whereas DAPI-labeled cells are shown in Fig. 4B, 4E,4H, 4K and 4N. In Fig. 4C, 4F,4I, 4L and 4O the pictures showing DAPI labeled cells and the DiI labeled cells merged together. Tregs in the peri-infarct zone were scarcely found on day 1 after the injection of labeled Tregs, but more abundantly found on day 4. Figure 4Q shows that Tregs significantly increased in number on day 4 and significantly decreased by day 7 by using one way ANOVA test (day 1 vs. day 4, *p*  =  0.007; day 1 vs. day 7, *p* <0.0001; day 4 vs. day 7, *p*  =  0.6).

### Adoptive transfer of Tregs attenuate left ventricular dysfunction

To examine whether Tregs exert a beneficial effect on cardiac remodeling, we transferred Tregs or PBS (as control) intravenously into mice with induced myocardial infarction.

**Figure 5:**
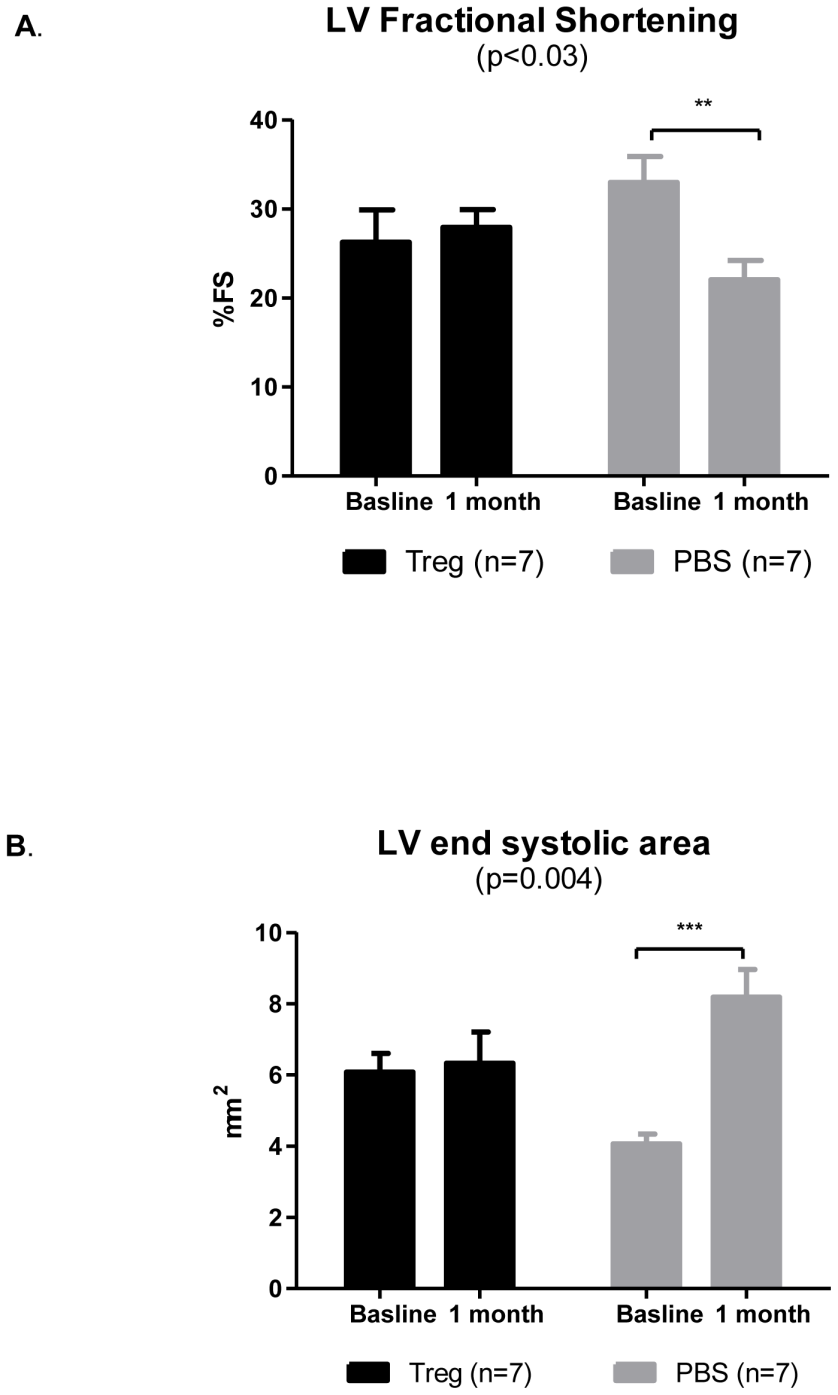
Adoptive transfer of Tregs improves cardiac function. (A) Treg-treated mice maintain fractional shortening, whereas control (PBS-treated) mice exhibit deterioration in this parameter. (B) Treg-injected mice exhibit no change in LVESA, whereas after 4 weeks the PBS-treated mice show a significant increase in LVESA compared to baseline. p based on Two-way repeated measures ANOVA; **p<0.007 and ***p<0.0005 based on 2-tail paired test.

Mice injected with Treg attenuated the typical course of dysfunction as assessed by Left ventricle fractional shortening (LVFS) compared with control (Fig. 5A, 5B). Control mice exhibited worsened remodeling process by fractional shortening (p<0.007). In addition, Treg-treated mice exhibit similar LV end systolic area (LVESA), whereas in the control mice there was a significant increase in this parameter (Fig. 5B, p  =  0.004).

Table 1 presents echocardiographic parameters of mouse LV function obtained by 2-D echocardiography 1 day after myocardial infarction (baseline) and 4 weeks after myocardial infarction was induced. Changes in echocardiographic variables are shown in Table 2. After 4 weeks, significant changes were evident in the LV end-diastolic dimension (LVEDD), LV end-systolic dimension (LVESD), LV end-systolic area (LVESA), FS and fractional area change (FAC), whereas the change in LV end-diastolic area was not significant. Control mice exhibited a significant increase in all the parameters. In contrast, Treg-treated mice showed a mild increase in LV dilatation indexes, including LV end-diastolic diameter (LVEDD) and LV end-diastolic area (LVEDA). We found a marked effect of Tregs injection in prevention of LV dilatation and in attenuation of LV dysfunction. We also observed a significant effect of interaction between the two groups.

**Table 1:** Comparison of left ventricular remodeling and function in Treg or PBS injected mice by 2-dimensional echocardiography 1 day after MI (baseline) and 4 weeks after first echo.

	Treg (n = 7)	PBS(n = 7)	p^1^ (repeated measures ANOVA)
**LVEDD, mm**			
Baseline	3.7±0.1	3.4±0.08	
4 weeks	3.9±0.2	4.2±0.14	
p^2^(paired t-test)	0.25	0.004	0.05
**LVESD, mm**			
Baseline	2.7±0.1	2.3±0.07	
4 weeks	2.8±0.2	3.2±0.19	
P^2^(paired t-test)	0.6	0.0009	0.006
**LVEDA, mm2**			
Baseline	11.05±0.5	10.32±0.5	
4 weeks	12.11±1.09	13.35±0.5	
P^2^(paired t-test)	0.4	0.004	0.17
**LVESA, mm2**			
Baseline	6.07±0.52	4.06±0.3	
4 weeks	6.3±0.8	8.18±0.7	
P^2^(paired t-test)	0.8	0.0005	0.004
**FS, %**			
Baseline	26.26±3.6	32.96±2.9	
4 weeks	27.93 ± 1.99	22.05±2.17	
P^2^(paired t-test)	0.7	0.007	0.03
**FAC, %**			
Baseline	44.9±4.2	60.3±2.8	
4 weeks	48.8±3.8	39.3±3.7	
P^2^(paired t-test)	0.5	0.0008	0.002

1. p in the right column reflect comparison of the differences between treatment and control groups over time. p based on 2-way repeated measures ANOVA.

2. p in the left columns are for the differences between baseline and 4-week measurements, based on paired t-test.

LVEDD  =  Left ventriculart end diastolic diameter; LVESD  =  Left ventricular end systolic diameter; LVEDA  =  Left ventricular end diastolic area; LVESA  =  Left ventricular end systolic area; FS  =  fractional shortening  =  [(LVDD (-) LVSD)/LVDD] x100; FAC  =  fractional area change  =  [(LVEDA-LVESA)/LVEDA]X100.

**Table 2:** Percentage of change^2^ from 1 day after MI (baseline) obtained by echocardiography 4 weeks after first echocardiography.

	Treg (n = 7)	PBS (n = 7)	p ^1^(unpaired t-test)
**LVEDD, mm**	5.6±4.5	21.06±4.9	0.04
**LVESD, mm**	4.89±7.5	41.48±6.75	0.003
**LVEDA, mm^2^**	10.8±10.2	31.25±7.9	0.14
**LVESA, mm^2^**	7.8±16.3	101.4±13.9	0.0009
**FS, %**	19.6±18.1	−31.7±5.8	0.02
**FAC, %**	14.1±12.4	−26.9±7.5	0.015

1. p based on unpaired t-test.

2. Percentage of change  =  (echo 2-echo 1)/echo 1x100.

Abbreviations as in Table 1.

### Transfer of Tregs reduces infarct size

**Figure 6:**
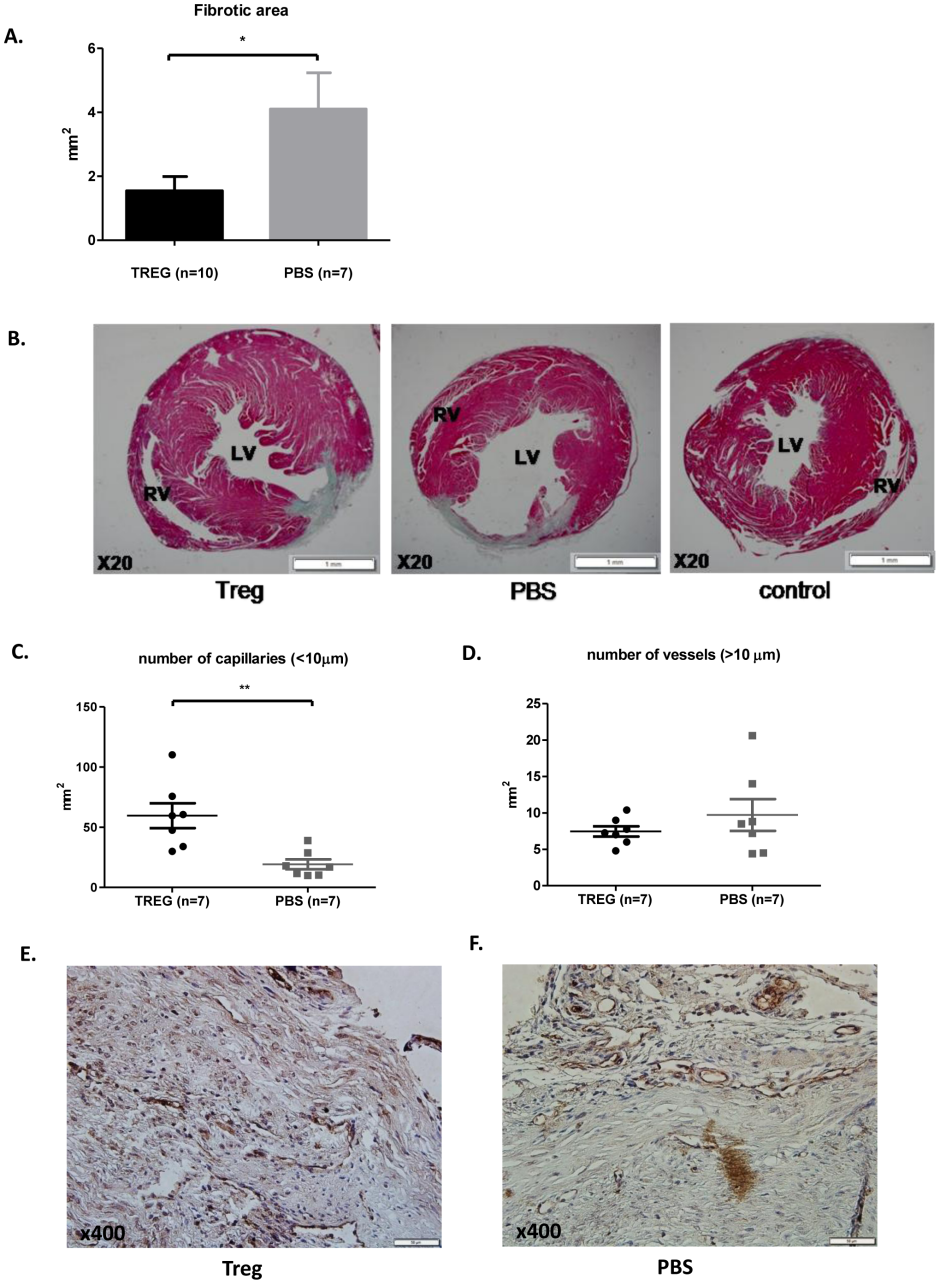
Adoptive transfer of regulatory T cells reduces infarct size. (A) Morphometry shows that treatment with Tregs reduces the proportion of fibrosis. (B) Representative sections of mouse heart treated with Tregs or PBS and labeled by Masson Trichrome staining at x20 magnification compared to control heat. (C) The number of capillaries was 2-fold greater in the Treg-treated group. (D) The number of vessels>10 µm was similar between the groups. (E–F) Serial sections were immunolabeled with antibodies against CD31 at x400 magnification. *p<0.02, **p<0.002 based on Mann-Whitney test.

Post-mortem heart sections were analyzed morphometrically 4 weeks following myocardial infarction induction. Masson's trichrome staining showed that Treg-treated hearts were relatively well preserved compared with the control group (*Fig. 6* *, p*  =  0.018 (1.55 ± 0.4 after Treg injection vs. 4.1 ± 1.1 after PBS injection).

A fibrotic area measured by morphometric analysis of the heart sections is shown in Figure 6A, and representative photographs of Treg-treated and PBS-treated hearts stained with Masson's trichrome are seen in Figure 6B.

### Effect of Tregs on vascularization

To determine the effect of Tregs transfer on vascularization, we harvested hearts 28 days after injection (after echocardiography imaging). CD31 staining showed that Treg-treated hearts was more than 2- fold greater than PBS treated hearts in a small capillaries (<10 µm) per mm^2^ (Fig. 6C, p = 0.002), while the number of vessels (>10 µm) per mm^2^ was similar between the two groups (Fig. 6D, p = 0.9), this finding is probably related to the number of Treg cells with well recognized angiogenic properties at the time of evaluation. Representative photographs of Treg-treated hearts and PBS treated hearts stained with CD31 are seen in Figure 6E and 6F.

### Treg depletion by PC61 antibodies

**Figure 7:**
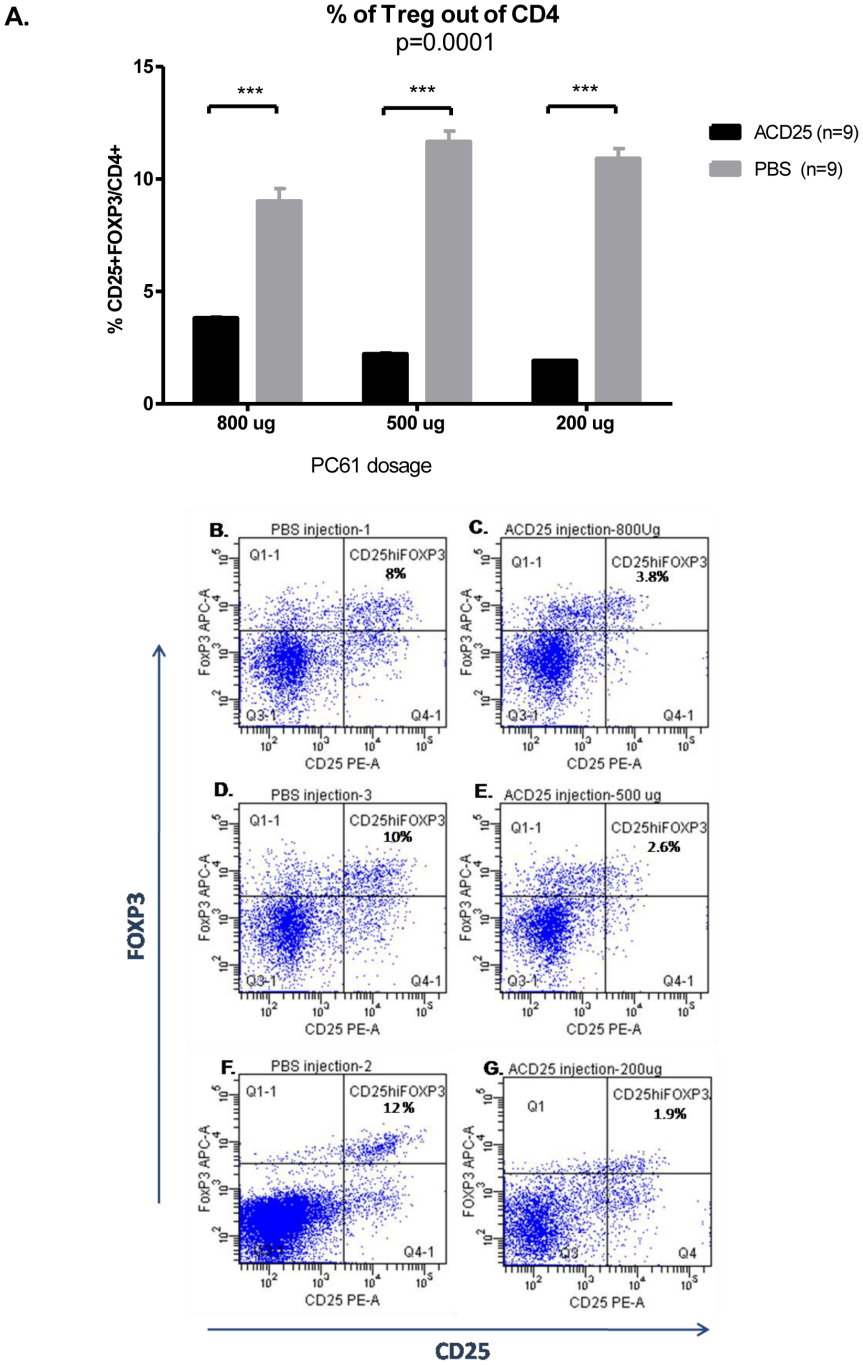
Anti-CD25 mAb (PC61) depletes CD4^+^CD25^+^FOXP3 cells. (A) The antibody dosage at three different ratios. Representative FACS picture shows the levels of CD25^hi^FOXP3 out of CD4^+^: (B), (D), (F) PBS injection group (C) ACD25 injection group: 800 µg (E) 500 µg and (G) 200 µg. p based on Two-way ANOVA; ***p<0.0001 based on Bonferoni's multiple comparison test.

To determine the role of Tregs in cardiac remodeling, we induced Treg depletion in mice one day before myocardial infarction induction. The antibody dosage was tested at three different ratios (Fig. 7A) to determine the correct dose that deplete Tregs. Consistent with previous reports [23]–[24], the results shows that lowerlevels of ACD25 induce a more potent deletion of Tregs markers. The observed variability is due to technical variability in the staining, as well as normal biological variation between different mice. It appears that although mice are age and sex matched, differences are noticed in vivo when antibody activity is sampled. To our experience, this variability is not surprising and we overcome it by repeating also in vivo studies several times.

Thus, in order to deplete Treg cells, 200 µg of anti-mouse CD25 monoclonal antibody clone PC61 were administered intraperitoneally starting on day 1 prior to induction of myocardial infarction. Representative FACS picture shows the levels of FOXP3CD25hi out of CD4+ cells (Fig. 7B–7G).

Numerous previously published studies have used these methods [25]–[27]. The depleting antibody has a different epitope than used in the flow cytometric assay [19].

In many reports, Tregs were shown to suppress immune responses via IL-10 production [28]–[29]. One study demonstrate lower levels of IL-10 in the corneas of Treg-depleted WT mice as compared with that in undepleted WT animals [30]. Another study we performed showed lower levels of IL-10 mRNA expression in Treg depleted mice as compared to the control group [31]. This may explain the issue that a reduced number of Treg cells demonstrate reduced Treg functional capacity.

### Treg ablation does not influence remodeling after experimental infarction

**Figure 8:**
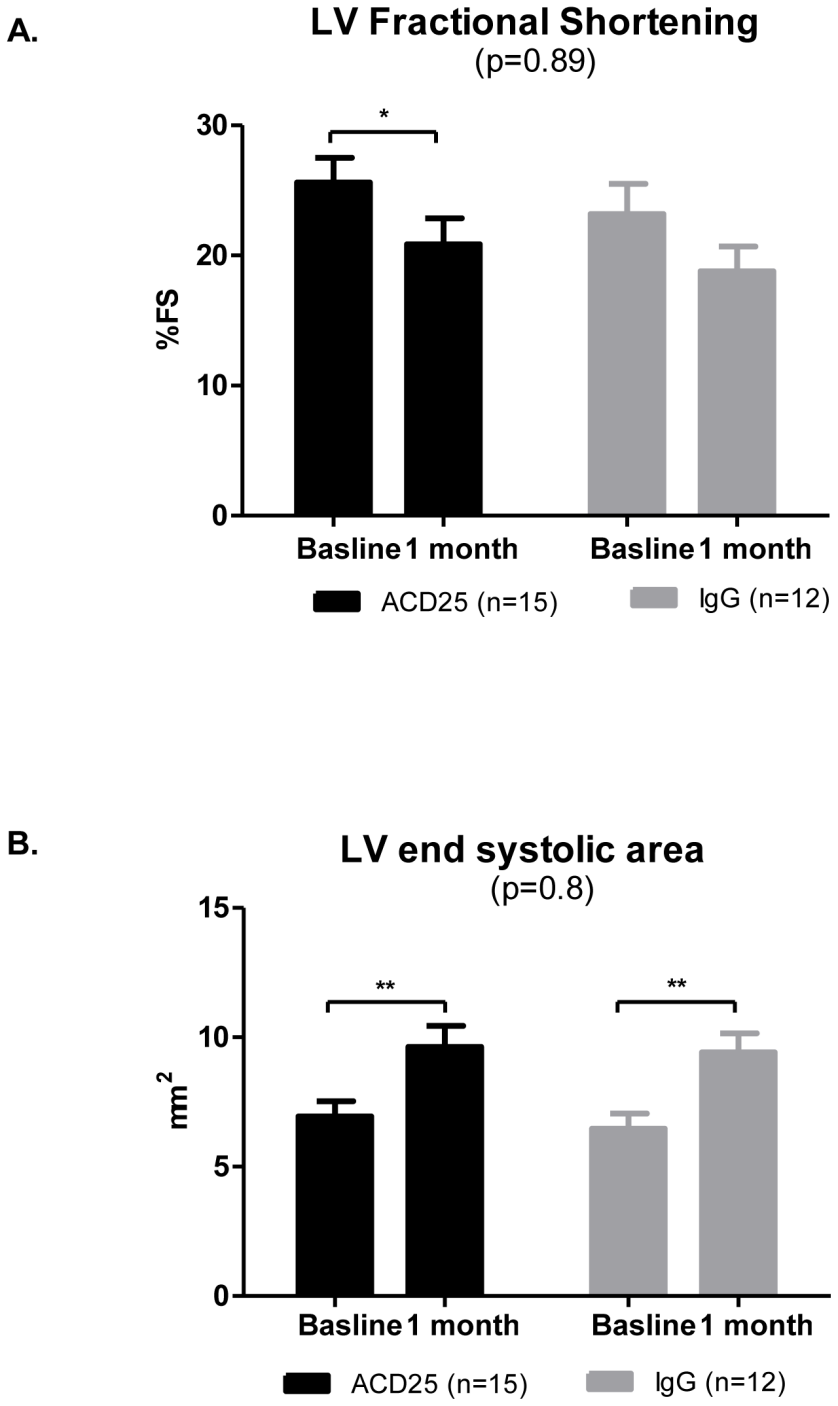
No effect on cardiac function after Tregs depletion. (A) ACD25 mAb or control (IgG isotype control) treated mice exhibit deterioration in FS parameter. (B) ACD25 mAb or control (IgG isotype control) treated mice exhibit significant increase in LVESA after 4 weeks compared to baseline. p based on Two-way repeated measures ANOVA; *p<0.02 and **p<0.002 based on 2-tail paired test.

No difference in echocardiography parameters was observed in the PC61 mAb injection group as compared with rat IgG isotype control group on day 28 after ischemia induction (Fig. 8A and 8B). Table 3 presents a comparison of mouse LV function obtained by 2-D echocardiography 1 day after myocardial infarction (baseline) and 4 weeks after myocardial infarction was induced. No differences were evident after comparing remodeling parameters between study groups LV. Changes in echocardiography variables are shown in Table 4.

**Table 3:** Comparison of left ventricular remodeling and function in ACD25 or IgG injected mice by 2-dimensional echocardiography 1 day after MI (baseline) and 4 weeks after first echo.

	ACD25(n = 15)	IgG (n = 12)	p ^1^(repeated measures ANOVA)
**LVEDD, mm**			
Baseline	3.79±0.13	3.7±0.1	
4 weeks	4.34±0.17	4.2±0.13	
P ^2^(paired t-test)	0.0174	0.0035	0.97
**LVESD, mm**			
Baseline	2.843±0.15	2.852±0.14	
4 weeks	3.46±0.19	3.46±0.17	
P ^2^(paired t-test)	0.0175	0.0094	0.99
**LVEDA, mm2**			
Baseline	12.4±0.63	11.81±0.5	
4 weeks	15.4±1.09	14.92±0.7	
P ^2^(paired t-test)	0.025	0.0016	0.92
**LVESA, mm2**			
Baseline	6.95±0.58	6.463±0.6	
4 weeks	9.633±0.81	9.42±0.7	
P ^2^(paired t-test)	0.0121	0.0051	0.813
**FS, %**			
Baseline	25.62±1.89	23.18±2.34	
4 weeks	20.87 ± 1.98	18.779±1.92	
P ^2^(paired t-test)	0.094	0.16	0.895
**EF, %**			
Baseline	50.51±3.072	46.39±3.912	
4 weeks	42.047±3.316	38.61±3.44	
P ^2^(paired t-test)	0.0715	0.092	0.88
**LVEDV, mm3**			
Baseline	63.2±5.03	58.94±3.9	
4 weeks	88.15±8.8	81.97±5.9	
P ^2^(paired t-test)	0.004	0.009	0.85
**LVESV, mm3**			
Baseline	32.73±4.06	32.23±3.603	
4 weeks	52.67±6.9	51.61±6.025	
P ^2^(paired t-test)	0.003	0.011	0.99
**EF, %**			
Baseline	50.51±3.072	46.39±3.912	
4 weeks	42.047±3.316	38.61±3.44	
P ^2^(paired t-test)	0.009	0.05	0.88

1. p in the right column reflect comparison of the differences between treatment and control groups over time. p based on 2-way repeated measures ANOVA.

2. p in the left columns are for the differences between baseline and 4-week measurements, based on paired t-test.

LVEDD  =  left ventricle end diastolic diameter; LVESD  =  left ventricle end systolic diameter; LVEDA  =  left ventricle end diastolic area; LVESA  =  left ventricle end systolic area; FS  =  fractional shortening  =  [(LVDD -LVSD)/LVDD]x100; FAC  =  fractional area change  =  [(LVEDA-LVESA)/LVEDA]X100; LVEDV  =  left ventricle end diastolic volume; LVESV  =  left ventricle end systolic volum; EF  =  ejection fraction.

**Table 4:** Percentage of change^2^ from 1 day after MI (baseline) obtained by echocardiography 4 weeks after first echocardiography.

	ACD25 (n = 15)	IgG (n = 12)	p ^1^ (unpaired t-test)
**LVEDD, mm**	15.4±3.9	16.15±5.6	0.91
**LVESD, mm**	23.95±6.2	24.4±8.23	0.96
**LVEDA, mm2**	25.54±7.2	30.02±10.5	0.72
**LVESA, mm2**	47.73±12.98	60.55±21.17	0.6
**FS, %**	−17.13±6.1	−14.37±9.156	0.8
**FAC, %**	−13.85±5.3	−13.91±63.57	0.99
**LVEDV, mm3**	44.11±11.62	49.13±19.5	0.8
**LVESV, mm3**	80.05±22.14	85.05±31.6	0.89
**EF, %**	−15.89±5.2	−13.58±8.074	0.8

1. p based on unpaired t-test

2. Percentage of change = (echo 2-echo 1)/echo 1x100.

Abbreviations as in Table 3.

### Tregs ablation does not alter infarct size

**Figure 9:**
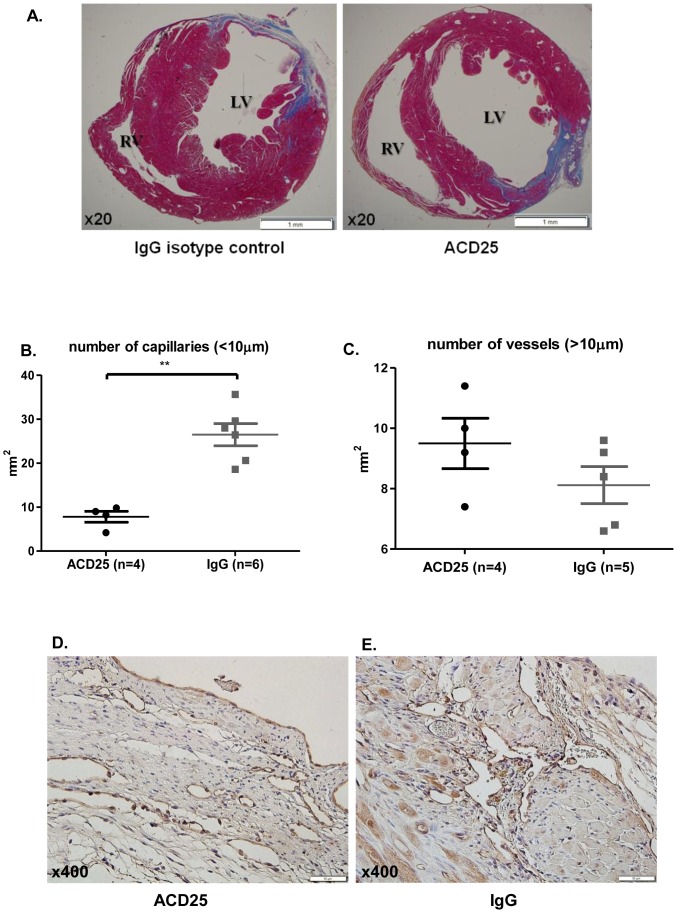
No effect at infarct size after Tregs depletion. Representative sections of mouse heart treated with (A) IgG isotype control or PC61 mAb and labeled by Masson Trichrome staining at x20 magnification. (B) The number of capillaries was 2-fold lower in the Treg ablation group. (C) The number of vessels>10 µm was similar between the groups (D–E) Serial sections were immunolabeled with antibodies against CD31 at x400 magnification. **p<0.001 based on Mann-Whitney test; n(ACD25)  = 4; n(IgG) = 6.

Post-mortem heart sections were analyzed morphometrically 4 weeks following myocardial infarction induction. Masson's trichrome staining confirmed no significant differences in infarct size between both groups (Fig. 9A). Representative photographs of ACD25 and IgG-treated hearts stained with Masson's trichrome are seen in Figure 9.

### Treg ablation dose influence Vascularization

To determine the effect of Tregs ablation on vascularization, we harvested hearts 28 days after injection (after echocardiography imaging). CD31 staining showed that ablation of Tregs resulted in a 2-fold reduction in small capillaries (<10 µm) per mm^2^ as compared to IgG treated mice (Fig. 9B, p = 0.0095), however the number of vessels (>10 µm) per mm^2^ was similar between the two groups (Fig. 9C, p = 0.8). Representative photographs of Treg ablation and IgG treated hearts stained with CD31 are shown in Figure 9D and 9E.

## Discussion

Upon ischemic cardiac damage, cardiac pro-inflammatory cytokines are upregulated following invasion of leukocytes and subsequently of lymphocytes. The cellular effectors and endogenous molecular signals implicated in suppression and containment of the inflammatory response in the infarcted heart are summarized in a recent review [32].

Several studies, demonstrate that patients with acute coronary syndromes with evident cardiac ischemia, exhibit reduced numbers and functional compromise of their peripheral Tregs [33]–[35], [14]. Two recent studies showed that Tregs play a beneficial role in the process of cardiac remodeling after myocardial infarction. The first of these reports showed that adoptive transfer of Tregs could attenuate cardiac dysfunction and fibrosis, however the sample sizes were very small [36]. Tang et al. [16] demonstrated that Tregs exhibit protective effects on LV structure and function in a rat model of myocardial infarction. In this paper the authors further showed that transfer of Tregs in the same model can reduce cardiac hypertrophy and interstitial fibrosis compared to sham-operated controls, and that these beneficial effects are probably achieved by various mechanisms, including modulation of inflammatory responses, inhibition of cytotoxic T-cell responses, and direct protection of cardiomyocytes against apoptosis.

The data obtained in the present study sheds additional light on the kinetics of peripheral splenocyte and local Treg accumulation in the infarcted heart. Furthermore, we provide additional support for a beneficial role of Treg transfer after experimentally induced myocardial damage. We have demonstrated the effects of Tregs in a model of myocardial infarction in mice. Compared with rats, mice provide better tools for the study of immune markers. We showed that compared with sham-operated mice, the levels of CD4^+^CD25^+^FOXP3 cells among the splenocytes of mice undergoing LAD ligation are significantly increased (*p*  =  0.02) as soon as 1 day post the procedure.

The absence of lymphocytes in the normal heart [22], along with our observation thatTregs exist in infarcted hearts, suggests that these cells migrate to the injured tissue, most likely in response to the injury, and may have an important role in controlling the local inflammation that influences remodeling.

We also studied the functional suppressive properties of Tregs from mice with induced myocardial infarction and show for the first time that Tregs isolated from mice after experimental infarction are dysfunctional in their suppressive properties. Collectively these results may suggest that experimental myocardial infarction alters in systemic Treg pool that could influence remodeling.

This finding apparently contrasts a reduction reported in Treg functional properties in patients with acute coronary syndromes, some of whom had sustained myocardial infarction [37]. Those patients, however, were found to have extensive coronary atheroma, whereas the mice used in that experiment study were not atherosclerotic [38]–[40].

Tregs were recently shown to be major cerebroprotective modulators of post-ischemic inflammatory brain damage targeting multiple inflammatory pathways [41]. Another study demonstrated that trafficking of T lymphocytes, particularly Tregs, is increased during healing that follows kidney ischemia-reperfusion injury [42]–[44]. These findings led us to hypothesize that transfer of Tregs might partially salvage ischemic damage in the heart. We therefore performed assays of adoptive transfer, as carried out previously in models of experimental autoimmune encephalomyelitis and diabetes [45], [39], [37]. Use of a mouse model of myocardial infarction allowed us to demonstrate that Treg improved myocardial performance after experimentally inducted infarction. Compared with PBS treatment, Treg transfer resulted in a significant reduction in infarct size. We validated this finding by separately measuring the size of the fibrotic areas and by echocardiographic measurements that showed prevention of LV dilatation and attenuation of LV dysfunction.

We were also able to show that Tregs migrate to the injured area, reaching a peak on day 4 after cell transfer. Migration of Tregs to the infarcted zone may control local inflammation by attenuating damage mediated both by T cells and by antigen-presenting cells, thus harboring protective effects on the damaged myocardium.

However, it appears that whereas delivery of exogenous Tregs attenuates remodeling, Treg ablation with monoclonal antibodies to CD25 was did not aggravate remodeling as infarct size and echocardiographic parameters remained unchanged. It thus appears that a threshold level of Tregs is sufficient to maintain the cardiac reparative process, and further exogenous delivery of these cells can aid in attenuating remodeling. If provision of Tregs does not produce the opposite effect of Treg depletion, there is no linear association between systemic Treg levels and the extent of protection against ischemia induced damage.

In conclusion, our study demonstrates that after experimental heart injury, Treg pool is altered in number and in function. Transfer of exogenous Tregs improves remodeling whereas their ablation does not influence the process. If further confirmed in humans these findings may be harnessed for developing novel modalities to attenuate cardiac damage after myocardial infarction.
